# Adipose tissue inflammation contributes to body weight loss induced by experimental chronic food allergy in mice

**DOI:** 10.1186/2045-7022-1-S1-O28

**Published:** 2011-08-12

**Authors:** Denise Carmona Cara, Luana PA Dourado, Mria LM Noviello, Debora M Alvarenga, Gustavo B Menezes, Adaliene VM Ferreira, Danielle G Souza

**Affiliations:** 1 Federal University of Minas Gerais- UFMG, Department of Morphology, Belo Horizonte, Brazil; 2 Federal University of Minas Gerais- UFMG, Morphology, Belo Horizonte, Brazil; 3 Federal University of Minas Gerais- UFMG, Basic Nursing, Belo Horizonte, Brazil; 4 Federal University of Minas Gerais- UFMG, Department of Microbiology, Belo Horizonte, Brazil

## 

Food allergy affects approximately 5% of children and 3% of the adult population in the western world. This disease is the manifestation of an abnormal immune response to antigens introduced into the organism orally and it is often mediated by IgE. Our group developed a chronic mouse model for the food allergy and one of the most remarkable alterations observed is a loss of body weight. However, the disturbances that trigger this loss of body weight are not clear. Thus, the purpose of this study was to investigate the mechanisms involved in weight loss of mice with ovalbumin-induced food allergy. With this purpose, BALB/c mice were subcutaneously sensitized with ovalbumin in aluminum hydroxide and challenged with the antigen containing diet for 7 days. The allergic mice showed significant weight loss with loss of adipose tissue, although it was not observed a reduction in food intake. These mice demonstrated adipose tissue inflammation characterized by increased leukocyte recruitment (visualized by intravital microscopy) and infiltration of mast cells, macrophages and regulatory T cells in the stroma. Moreover, we demonstrated high concentrations of TNF-α, IL-6, IL-10 and the chemokine MCP-1/CCL-2 in this tissue. The metabolic changes in adipose tissue of allergic animals were represented by increased glucose uptake and lipolysis in adipocytes, resulting in atrophy of these cells. Changes were also seen in systemic metabolism characterized by decreased serum concentrations of glucose, triglycerides, total cholesterol and free fatty acids in allergic mice. Based on our results, we conclude that food allergy induces adipose tissue inflammation by producing mediators that lead to atrophy of this tissue. The decrease in adipose tissue mass has systemic consequences and results in loss of body weight.

